# T39. NEURAL MECHANISMS OF METABOTROPIC GLUTAMATE RECEPTOR 3 MEDIATED ENHANCEMENT OF SYNAPTIC PLASTICITY AND COGNITION

**DOI:** 10.1093/schbul/sby016.315

**Published:** 2018-04-01

**Authors:** Branden Stansley, Max Joffe, Samantha Yohn, Craig Lindsley, Colleen Niswender, P Jeffrey Conn

**Affiliations:** 1 Vanderbilt University; 2 Vanderbilt University Medical Center

## Abstract

**Background:**

The group II metabotropic glutamate receptor 3 (mGlu3) is an emerging therapeutic target for schizophrenia, as research has demonstrated a link between mutations in the human gene encoding for mGlu3, GRM3, and clinical diagnosis of schizophrenia. Schizophrenia is known to be accompanied by debilitating cognitive impairments that negatively impact the overall quality of life of the patient. While current pharmacological therapeutics mainly target the positive symptoms, cognitive symptoms are often not effectively treated. Our recent discovery that mGlu3 and mGlu5 can act as signaling partners to modulate synaptic plasticity in the prefrontal cortex led us to hypothesize that mGlu3 may subserve similar functions to those of mGlu5 during hippocampal synaptic plasticity and hippocampal-dependent behaviors.

**Methods:**

We directly tested this hypothesis using acute slice electrophysiology to investigate basal synaptic transmission as well as long-term plasticity in hippocampal slices. To test cognition, the associative fear learning behavioral assay, termed trace-fear conditioning, was used. C57bl/6 mice or CaMKII-cre;mGlu5-/- mice were used in all studies.

**Results:**

We report that mGlu2/3 activation enhances hippocampal theta-burst (TBS)-induced LTP but was without effect on group I mGlu agonist-induced LTD The group II mGlu agonist enhancement of TBS-LTP was blocked by antagonists of mGlu3 or mGlu5.

We next tested downstream mechanisms of group II mGlu induced LTP by chemically activating LTP with the group II agonist LY379268 in combination with selective antagonists. We verified the LTP was induced by mGlu3 activation but not mGlu2 using selective negative allosteric modulators of each subtype. Furthermore, mGlu5 negative allosteric modulation with MTEP blocked mGlu3-LTP, and conversely the mGlu5 positive allosteric modulator, VU0092273, enhanced mGlu3-LTP. The cannabinoid receptor type 1 antagonist AM251 was also capable of blocking mGlu3-LTP, suggesting cannabinoid signaling mechanistically drives this LTP.

Having confirmed a role for mGlu5 in the mGlu3-LTP, we next verified that postsynaptic mGlu5 located on pyramidal neurons was necessary for mGlu3-LTP by utilizing CaMKII-cre;mGlu5-/- mice. It was found that hippocampal slices from these mice showed no enhancement of LTP when LY379268 was bath applied alone or in combination with TBS-stimulation.

Behaviorally, we discovered that selective activation of mGlu3 by systemically injecting the group II mGlu agonist in combination with a selective mGlu2 negative allosteric modulator, VU6001966, causes an enhancement in the acquisition of trace-fear conditioning learning. This was also confirmed to be dependent on mGlu5 as both systemic pharmacological inhibition or genetic deletion of mGlu5 abolished this learning enhancement. Further testing of the ability of mGlu3 activation to augment other cognitive tasks is currently underway.

**Discussion:**

These results taken together demonstrate mGlu3 enhances hippocampal LTP and hippocampal-dependent learning through mechanisms that involve both mGlu5 and CB1 receptor activation. This work provides a basic biological mechanism and preclinical therapeutic validation for mGlu3 as a target for neurological disorders in which cognition is disrupted such as schizophrenia.

